# Evaluation of the effects of a drug with fiscalized substance dispensation, health education, and pharmacovigilance continuing education program in Colombia drugstores and drugstores/pharmacies: study protocol of a multicenter, cluster-randomized controlled trial

**DOI:** 10.1186/s13063-020-04481-1

**Published:** 2020-06-19

**Authors:** Mauricio Ceballos, Andrea Salazar-Ospina, Daniel Sabater-Hernández, Pedro Amariles

**Affiliations:** 1 Research Group on Pharmaceutical Promotion and Prevention, University of Antioquia, U.de.A. Calle 70 No 52-21, Medellin, Colombia; 2 Research Group on Pharmacy Regency Technology, University of Antioquia, Medellin, Colombia; 3 Pharmaceutical Care Research Group, Faculty of Pharmacy, University of Granada, Granada, Spain

**Keywords:** Dispensation, Health education, Pharmacovigilance, Drugstores, Pharmacies, Pharmacy staff, Fiscalized substances, Controlled drugs

## Abstract

**Background:**

Health disorders, due to the use of drugs with fiscalized substances, including controlled substances, have become a common problem in Colombia. Multiple reasons can help explain this problem, including self-medication, since access to these drugs may be easier. Also, there is a lack of knowledge that these drugs are safer than illicit drugs. The use of these drugs without a valid medical prescription and follow-up can have negative consequences such as drug abuse, addiction, and overdose, and eventually, have negative health consequences. Pharmacy staff is essential to both assure the correct drug use and minimize prescription errors to help outpatients have better management of their pharmacotherapy. For this reason, it is necessary to increase key competencies like knowledge, skills, and attitudes in the pharmacy staff of ambulatory (outpatients) pharmacies.

**Methods:**

This study is a prospective, cluster-randomized, parallel-group, multicenter trial of drugstores and drugstores/pharmacies (ambulatory pharmacies). The study is designed to determine the effectiveness of a drug with fiscalized substance dispensation, health education, and pharmacovigilance continuing education program in drugstores and drugstores/pharmacies. Pharmacy staff will be randomly selected and assigned to one of the study groups (intervention or control). The intervention group will receive a continuing education program for over 12 months. The control group will receive only general information about the correct use of complex dosage forms. The primary objective is to evaluate the effectiveness of a continuing education program to improve pharmacy staff competencies (knowledge, skills, and attitudes) to improve the ambulatory (outpatient) pharmacy services: dispensation, health education, and pharmacovigilance of drugs with fiscalized substances. The secondary outcomes include (a) processes associated with the management of drugs with fiscalized substances in drugstores and drugstores/pharmacies, including regulation compliance; (b) degree of implementation of ambulatory (outpatient) pharmacy services targeting these drugs in drugstores and drugstores/pharmacies; (c) patient satisfaction with such services; and (d) pharmacy staff satisfaction with the continuing education program.

**Discussion:**

This clinical trial will establish whether providing a continuing education program for the adequate utilization of drugs with fiscalized substances improves pharmacy staff competencies regarding these drugs.

**Trial registration:**

ClinicalTrials.gov NCT03388567. Registered on 28 November 2017. First drugstore or drugstore/pharmacy randomized on December 1, 2018.

**Protocol version:**

0017102017MC

## Background

Drugs with fiscalized substances, for example, benzodiazepines, tricyclic antidepressants, anxiolytics, antipsychotics, opioid analgesics, among others, are essential in various fields of medicine, such as pain treatment, obstetric emergencies, and mental disorders such as the treatment of drug dependence substances, psychiatry, and neurology. These substances are regulated by international conventions on drug control, recognizing their potential to be misused and cause harm, such as dependency syndrome and health disorders [1]. Drugs with fiscalized substances have a substantially high risk of causing a substance use disorder [2]. As a result, disorders due to drug abuse have become a common problem. Multiple reasons can help explain this problem, including self-medication, because access to these drugs may be more accessible. Besides, there is a lack of belief that these drugs are safer compared to illicit drugs [2]. Furthermore, there are failures in the surveillance and control of the processes; for instance, worldwide, it is common that many non-over-the-counter medications, including medications with controlled substances, are dispensed without a valid medical prescription even when it is mandatory [3].

Worldwide, there is evidence on the effectiveness and relevance of pharmaceutical interventions in combination with Information and Communication Technologies (ICT), continuing education programs, and network support between pharmacy staff and other health professionals on the use of drugs with fiscalized substances [4–7]. Competent pharmacists with positive attitudes, skills, and enough knowledge are crucial to the outcome of pharmaceutical care for patients. Pharmacists are required to improve the correct use of drugs, minimize medication errors, and help outpatients have better management of their pharmacotherapy. However, it is necessary to increase those competencies, especially in the correct use of drugs with fiscalized substances, including controlled drugs. These strategies are essential to improve collaboration and, consequently, to achieve optimal medication management. In this regard, the impact of continuing education in pharmacy staff has been described, focusing efforts on promoting the proper use of drugs, including opioids, antidepressants, and anxiolytics, and even preventing the abuse of these medications [8–12].

In the Colombian context, it is possible to consider the global concept of ambulatory (outpatient) pharmacies [13]. In this concept, it is possible to include (a) ambulatory (outpatient) pharmaceutical services (similar to the international concept of community pharmacies) which play a part in the care of patients affiliate to health system and (b) drugstores and drugstores/pharmacies, which may or may not play a part in the care of patients affiliate to the health system. Therefore, the correct homologation to community pharmacies would be ambulatory pharmaceutical services. However, the main concern regarding the correct use of drugs with controlled substances is found in the context of the drugstores and drugstores/pharmacies, and not in ambulatory pharmaceutical services.

Colombian drugstores and drugstores/pharmacies dispense drugs, medical products, drugs based on natural resources, cosmetics, and other products (e.g., toiletry products). These pharmacies are private healthcare establishments, and unfortunately, some non-over-the-counter medications are being dispensed without medical prescription. For example, in the case of antibiotics, a study found that 80.3% of the sales of antibiotics in drugstores and drugstores/pharmacies of Bogota dispensed without a valid medical prescription and so not fulfilling the current established legal requirements. Additionally, pharmacy staff is not sufficiently educated to manage and process these drugs. These situations contribute to the inappropriate use of antibiotics and are factors that induce antimicrobial resistance [14].

In the Colombian context, the pharmacy staff is a person who works within drugstores and drugstores/pharmacies with any of the following levels of training (professional, technological, or technical). Besides, there is the empirical profile of the Drug Dealer (a name given to those who do not have technical, technological, or professional training; they are also called Director of Drugstores). Furthermore, in the Colombian context, the pharmacy staff of drugstores and drugstores/pharmacies are frequently the first healthcare providers with whom a patient comes into contact before using a medication, principally because the ambulatory (outpatient) pharmacies are accessible. It is common for patients not to visit their doctors until their health problems worsen.

Further, some studies suggest that imposing an excessive workload on pharmacy staff reduces the time that they spend with each patient and leads to less control over medical prescriptions [15]. The capacity to improve therapeutic outcomes, patients’ quality of life, scientific advancement, and enhancement of our public health imperatives is dependent on specific competencies of the pharmacy staff [16]. For the World Health Organization (WHO) and Pharmaceutical International Federation (FIP), these competencies include knowledge, skills, and attitudes, which are revealed when they perform either a task or a job. Competencies could be achieved through education, training, and experience [17].

To the best of our knowledge, there is no information available from studies related to pharmacy staff competencies in the provision of outpatient pharmacy services: dispensing, health education, and related pharmacovigilance related to controlled substance drugs (including controlled drugs). However, some studies have evaluated the effect of educational interventions on the pharmacist to improve knowledge and practices in health problems in which the use of drugs with fiscalized substances is a therapeutic alternative, such as depression, anxiety, and substitution treatment of opioids [10, 18, 19].

Consequently, the implementation of a continuing education program through the integration of different methods and tools supported by ICT, such as a web-based social networking site and a virtual classroom, could favor access to relevant and quality information on health promotion, disease prevention, and the proper use of drugs with fiscalized substances (including controlled drugs). They facilitated the improvement of the labor competencies of the pharmacy staff, in addition to improving the satisfaction of patients and caregivers about the ambulatory (outpatient) pharmacy services offered in drugstores and drugstores/pharmacies.

## Objectives

The primary objective is to evaluate the effectiveness of a continuing education program to improve pharmacy staff competencies (knowledge, skills, and attitudes) to improve the ambulatory (outpatient) pharmacy services: dispensation, health education, and pharmacovigilance of drugs with fiscalized substances (including controlled drugs). The secondary objective is to characterize the processes associated with the utilization of drugs with fiscalized substances (including controlled drugs) and regulation compliance and the degree of implementation of ambulatory (outpatient) pharmacy services. The third objective is to evaluate the satisfaction of patients and caregivers with ambulatory (outpatient) pharmacy services. The fourth objective is to evaluate the satisfaction level of pharmacy staff with the provided educational program.

## Trial design

**Fig. 1:**
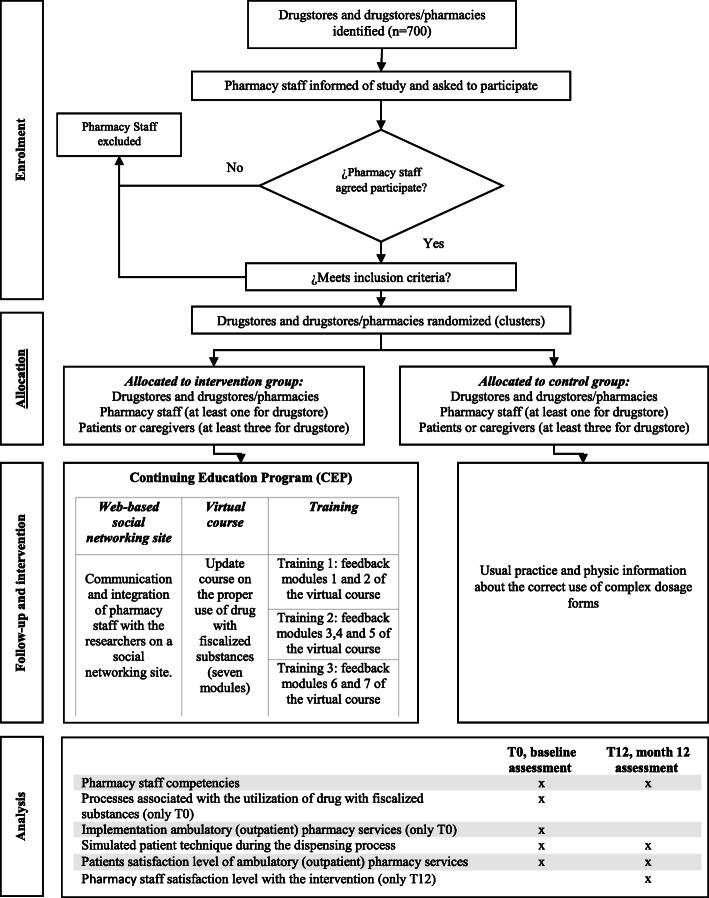
The continuing education program process and methodology

**Fig. 2:**
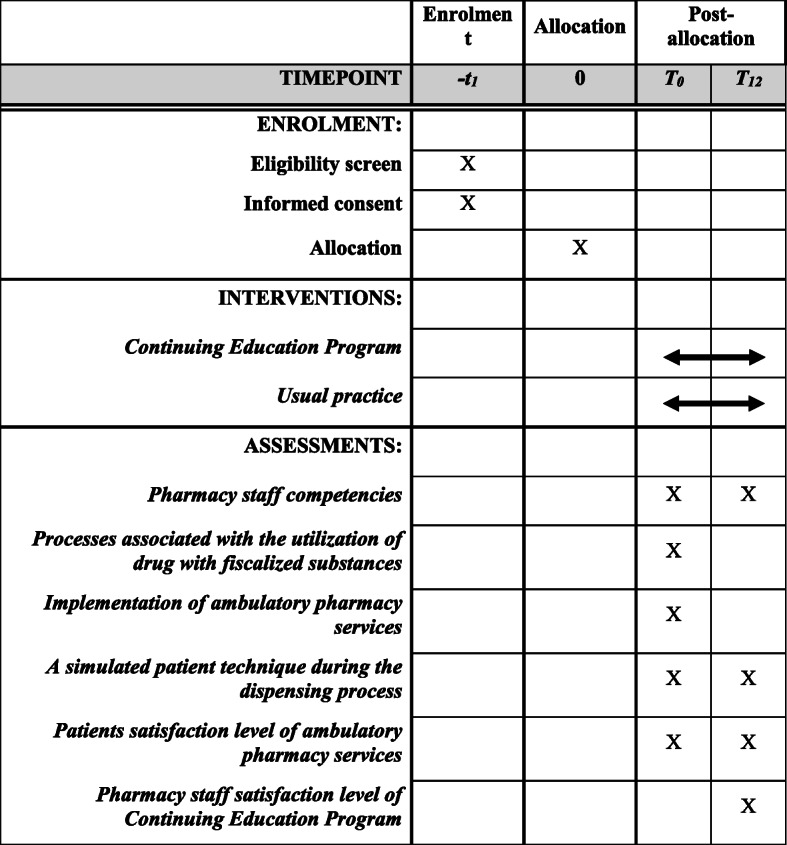
Description of the registration calendar, interventions, and evaluations

A multicenter, prospective, parallel-group, cluster-randomized controlled, superiority trial will be conducted (see Figs. 1 and 2). The study will be carried out over 12 months (1 year) of intervention (i.e., the continuing education program) delivery and follow-up. Drugstores and drugstores/pharmacies (the clusters) will be randomized at a 1:1 ratio to enroll in either the intervention group or the control group. The geography zone (neighborhoods) will be randomized stratified.

## Methods

### Study setting

The study will be conducted in drugstores and drugstores/pharmacies of Antioquia (one of the 32 Colombian departments), including the central city, Medellin, and the Metropolitan Area. Medellin is the capital of the department of Antioquia and has over 3.5 million inhabitants in 10 municipalities (Metropolitan Area of Medellín) by 2015. According to the projection by the United Nations, it is estimated that 4,389,585 people would inhabit the Metropolitan Area, 844,882 more people between 2010 and 2030, at a rate of 1% per year and with higher dynamics in the urban area [20].

Additionally, the number of drugstores and drugstores/pharmacies has been growing steadily. According to data of the study of characterization of the pharmaceutical sector in Colombia by 2014, carried out by the National College of Pharmaceutical Chemists of Colombia, which is the central organization of the professional pharmaceutical union in the country, there were more than 20,000 drugstores and drugstores/pharmacies [21].

### Eligibility criteria

#### Inclusion criteria

##### Drugstores and drugstores/pharmacies

From a list of 1500 drugstores and drugstores/pharmacies of Antioquia, we identified those that participated in a cross-sectional epidemiological study on the use of drugs with fiscalized substances carried out in 2016 [22]. Drugstores and drugstores/pharmacies will be eligible for inclusion in the study if they are registered with the delegated entity for surveillance, which is located in Medellín and the Metropolitan Area.

##### Pharmacy staff

The study will recruit at least one pharmacy staff member from each drugstore or drugstore/pharmacy (professional, technological, technical, or Drug Dealer). To be included in the study, the pharmacy staff will have to meet the following criteria: (a) work in the drugstores or drugstores/pharmacies and (b) have access to a computer and the Internet.

##### Patients or caregivers

The study will recruit at least three patients or caregivers from drugstores or drugstores/pharmacies and will assess whether each patient meets all the inclusion criteria. The inclusion criteria will be the following: (a) patients or caregivers should be at least 18 years old, (b) they must be at the drugstores or drugstores/pharmacies at the time of being interviewed by the researcher, and (c) dispensing at least one drug with or without a medical prescription.

#### Exclusion criteria

Pharmacy staff and patients/caregivers that refuse to sign the consent form are not included (it is necessary to obtain the consent before any study-related procedures were conducted).

### Sample size

A study carried out in Spain evaluated the competences pharmacists, principally the knowledge before and after an educational intervention facilitated by pharmacists, finding that the intervention increases in 37% the knowledge of the pharmacists who attended the course [23]. Therefore, the continuing education program is expected to improve the pharmacy staff competencies in the intervention group at least 30% and in the control group by 15% (less in improving or increasing competencies). With a confidence level of 95% and a power of 80% and superiority design, it was calculated that it is necessary to include at least 121 drugstores or drugstores/pharmacies in each group. To cover losses to follow-up, the sample was increased by 10%, increasing the sensitivity for the fulfillment of secondary objectives; the size of the final sample will be 135 drugstores or drugstores/pharmacies for each group (1:1 ratio).

### Recruitment

#### Drugstores or drugstores/pharmacies and pharmacy staff

We will invite the pharmacy staff of 700 drugstores and drugstores/pharmacies to participate in the study. The researchers will explain to the pharmacy staff the objectives and the study design in meetings where they will accept their participation. Besides, all drugstores and drugstores/pharmacies will be visited by a pharmacist (no researcher) to obtain their written consent, between December 2018 and January 2019. The drugstores and drugstores/pharmacies that do not attend the meetings will be called by phone by a researcher to invite them to the rehearsal, and then they will be visited. If neither pharmacy staff of drugstores and drugstores/pharmacies do not agree to participate in the trial, they will be replaced by another from the same geographic area. Pharmacy staff will not receive any economic compensation for participating in the study. The researchers will ensure that the data collected is anonymous and strictly confidential.

The inclusion and exclusion criteria of pharmacy staff that accept to participate in the study will be evaluated. Some of their demographic and academic characteristics will be recorded: age, sex, highest educational level, year of pharmacist title (if applicable), university or educational institution where degree was obtained (if applicable), function in the pharmacy (owner/worker), hours worked per week, continuing education in recent years, the intensity of continuing education, time of experience as a pharmacy staff, and if they currently live in the same neighborhood as the drugstores or drugstores/pharmacies where they are employed.

#### Patients or caregivers

At least three patients or caregivers from each drugstore or drugstore/pharmacy will be recruited who had previously been dispensed with medication by the pharmacy staff of each drugstore or drugstore/pharmacy. The researchers will inform the potential candidates (patients or caregivers) about the study and will ask for their authorization to participate; they will be registered and will sign a consent form. The variables related to the objectives of the study in the case of the patients who agree to participate will be assessed. The patients will not receive any compensation for participating in the study.

One of the researchers will make visits to the pharmacy staff. The visits will be organized according to the geographical distribution where the drugstores and drugstores/pharmacies are located. The researcher will enter the drugstores or drugstores/pharmacies and remain there for as long as necessary to interview a minimum of 3 patients or caregivers who enter the drugstores or drugstores/pharmacies requesting the drug dispensing process. Patients or caregivers will be selected in order of arrival or admission to the drugstores or drugstores/pharmacies. The researcher will approach the patient or caregiver and invite them to participate in the study. If the patient or caregiver agrees, they will sign the informed consent, and they will be interviewed to complete the measurement questionnaire. There will be no selection criteria related to the characteristics of the patient or caregiver, nor of the medications dispensed.

### Allocation

Group allocation (the intervention group or the control group) will be blinded and will be determined using a computer-generated random number table and placed in numbered, designed by a person external to the study and otherwise unrelated to it using Microsoft Excel® (version 2010, Microsoft ®).

### Blinding

Blinding of pharmacy staff is not possible because of the nature of the intervention. Patients and caregivers will be able to be blinded because they will not receive given details of the intervention.

### Interventions

#### Intervention group

The continuing education program is made up of 3 different and independent actions that will be executed transversely during the duration of their implementation and follow-up (12 months). These actions will be applied at different times, as described in Fig. 1. Next, we give details of each of the actions.

##### Web-based social networking sites and access to a virtual classroom

The integration of pharmacy staff into a web-based social networking site and access to a virtual classroom can favor access to relevant and quality information on health promotion, disease prevention, and correct use of drugs. The access and interactivity in the web-based social networking site will be continuous 24 h 7 days a week during the 12 months that the intervention lasts. Web-based social networking will be designed and built according to current trends, to develop a highly usable and accessible site based on the requirements and functionalities of an expert panel. Some of the characteristics and functionalities of the web-based social networking site will be as follows:

- Pharmacy staff will access from personal computers (PC) and mobile devices with a touch screen.
- Pharmacy staff will be able to describe their status; to establish communication with other pharmacy staff, the administrators of the platform, researchers, and invited experts; to establish their geographical location; to create events; and to upload images, videos, and links from other pages, resources in the web, or social networking sites such as YouTube, Facebook, Twitter, and Instagram.
- Pharmacy staff will comment on all materials uploaded to the platform by different users (pharmacy staff, researchers, or administrators of the platform). Additionally, they will have access to video tutorials, frequently asked questions (FAQ), comments, forums, opinion polls, and downloadable document libraries.
- A gamification system must be introduced to encourage its use with which all user actions (point system). The gamification system will consist of a pharmaceutical marketing strategy to generate value to the person who is participating in a course so that they are motivated to develop and finish it. For example, generate their content (images, photographs, and videos). Also, they will be linked with content from other social networking sites such as YouTube, Facebook, Twitter, and Instagram to interact with other users.
- Pharmacy staff will be accessing a virtual classroom of an update course for the proper utilization of drugs with fiscalized substances.

##### Virtual course

The virtual course will be composed of 7 modules. The study time for each module will require an average of 20 h of study (ten synchronous hours and ten asynchronous hours). Each module will be structured as follows: (a) initial evaluation,( b) development of themes and content, and (c) final evaluation. The virtual course will be designed using Moodle 3.3.2+, which is a Learning Management System (LMS) for the development, administration, and documentation of learning. The virtual course will have a combination of different multimedia tools such as audios (locutions), videos with experts, animated videos, animations, videocasts, infographics, and interactive tests with drag and drop exercises, association, completion, and multiple selection responses.

The development of the virtual course will not be carried out in a synchronized manner among all subjects. Therefore, the course will be carried out in a staggered way; that is, the seven modules will be activated systematically 1 to 7, on average, every 45 to 60 days. These modules constitute a refresher course in knowledge, and those who complete all the modules of the virtual course will be certified by the University of Antioquia. It will be established as an actualization course.

For the development and construction of the virtual course, a descriptive observational study was conducted in 2016, seeking to characterize the needs and perceptions of information and continuing education to favor the proper use of drugs with fiscalized substances [22]. The current Colombian regulations related to the competencies necessary for work performance have been reviewed (Colombian regulations focused on the classification of labor competencies for the performance of ambulatory pharmaceutical services and drugstores and drugstores/pharmacies). Subsequently, the topics and contents were selected and supported with a review of the literature to build the seven modules: (1) pharmaceutical terminology and basic pharmacology, (2) pharmaceutical legislation, (3) technical and administrative management of the pharmacy, (4) communication and health education from the pharmacy, (5) good practices of dispensation and proper utilization of drugs, (6) pharmacovigilance from the pharmacy, and (7) pharmaceutical actuation of some common diseases in the pharmacy and in which drugs with fiscalized substances are used (depression, anxiety, sleep disorders, and pain management). Finally, this information was socialized and reviewed with a panel of experts. Table 1 describes the objectives of the modules of the course for the proper utilization of drugs with fiscalized substances.

**Table 1:** Objectives of the virtual course modules for the proper utilization of drugs with fiscalized substances

Module	Objectives
Module 1. Pharmaceutical terminology and basic pharmacology	1. Identify the different concepts and terms associated with pharmacology and pharmacotherapy. 2. Understand the concepts of the processes covered by pharmacokinetics (release, absorption, distribution, metabolism, and excretion) and pharmacodynamics (action and effect). 3. Know the different types of drug interactions and their implications on the patient.
Module 2. Pharmaceutical legislation	1. Know the essential and practical concepts of legislation in Colombia. 2. Differentiate the standards corresponding to pharmaceutical products, establishments, and pharmaceutical services. 3. Identify the different norms that regulate the use of controlled drugs and drugs with fiscalized substances in Colombia.
Module 3. Technical and administrative management of the pharmacy	1. Recognize the appropriate mechanisms for the selection and purchase of drugs and pharmaceutical products, contributing to the proper management of inventories. 2. Identify strategies for the reception and storage of drugs and pharmaceutical products under optimal conditions of temperature, humidity, and cleanliness. 3. Know the recommendations for inventory management and management.
Module 4. Communication and health education from the pharmacy	1. Know the strategies for communication and dissemination of information on medications and health issues, as well as the tools and skills to carry out health education activities to patients. 2. Identify some of the recommendations for carrying out communication and health education activities for special populations (pediatrics, pregnancy, and the elderly).
Module 5. Good practices of dispensation and proper utilization of drugs	1. Know the necessary recommendations for the correct practice of dispensation medications. 2. Relate the proper use of drugs, the aspects involved in said use, and the responsibility of the pharmacy staff.
Module 6. Pharmacovigilance on the pharmacy	1. Learn the basic knowledge of pharmacovigilance, as well as the strategies for causality analysis of adverse drug effects. 2. Know the recommendations to implement a pharmacovigilance program from a pharmacy.
Module 7. Pharmaceutical actuation of some common diseases in the pharmacy (depression, anxiety, sleep disorders, and pain management)	1. Identify recommendations and pharmacotherapeutic strategies for pain management. 2. Differentiate recommendations and pharmacotherapeutic strategies for the control of depression, anxiety, sleep disorders, and other mental disorders. 3. Recognize the risks associated with improper use and abuse of drugs with fiscalized substances.

##### Pharmacy staff training

All pharmacy staff of the intervention group will be invited to participate in three face-to-face meetings as a complement to the virtual course for the proper utilization of drugs with fiscalized substances. Each meeting will last 4 h, for a total of 12 h of face-to-face training. Each pharmacist will make their presentation according to the topics programmed in each training meeting: (a) training 1, feedback from modules 1 and 2 of the virtual course; (b) training 2, feedback from modules 3, 4, and 5 of the virtual course; and (c) training 3, feedback from modules 6 and 7 of the virtual course. Lectures will be prepared in PowerPoint presentations, videos, and practical activities such as role-playing and case presentation. The pharmacists who will conduct the face-to-face meetings are professional pharmacists, with experience in teaching and research in the specific topics and a strong background in clinical and therapeutic pharmacy. The professional pharmacists are also co-authoring the virtual course modules.

#### Control group

Due to the nature of the study, there will be no blinding since the pharmacy staff of drugstores and drugstores/pharmacies that meet the inclusion criteria will be informed about the study and will register after their signed authorization. Pharmacy staff in the control group will receive written material on the correct use of complex pharmaceutical forms, with information that focuses on the importance of providing this information to patients and caregivers to achieve the goals of treatment. Randomization will take place at baseline. The written material will be delivered during the follow-up time.

### Criteria for discontinuing

When pharmacy staff leave or change their place of work to the original one where they were initially randomized in the study or decide voluntarily to withdraw from the study, it will be considered as a criterion for discontinuing.

### Strategies to improve adherence to intervention protocols

The pharmacy staff in both groups will receive phone calls from one of the researchers to encourage compliance with the protocol. The pharmacy staff of the study will not be blinded to the assignment of the intervention, and the same researcher will visit both groups. The researcher will visit all drugstores and drugstores/pharmacies to train and provide technical support to the pharmacy staff of the intervention group in the access and use of the virtual classroom and the virtual course. During the 12 months that the monitoring and intervention will last, one of the researchers will be available to receive telephone calls to solve doubts and academic concerns and provide technical support related to the virtual classroom and the virtual course. Telephone calls have several purposes: (1) accompany pharmacy staff during the study and promote adherence to the continuing education program for their development, (2) accompaniment and technical support in the use of the educational platform, and (3) promote communication to invite pharmacy staff to face-to-face training sessions to be scheduled to complement their training.

### Outcomes

Pharmacy staff competencies (knowledge, skills, and attitudes) will be assessed using two self-applied questionnaires. The measurements will be performed at baseline (T0) before the intervention and 12 months (1 year) after completing the follow-up (T12). Additionally, the simulated patient technique will be used to evaluate the skills and attitudes of the pharmacy staff in the dispensation and the information supply for the correct use of drugs (T0 and T12).

### Secondary outcomes

The following are the secondary outcomes of the study:

- Characterization of the processes in the drugstores and drugstores/pharmacies. The measurements will be performed only at baseline (T0) before the intervention.
- Implementation of the ambulatory (outpatient) pharmacy services in the drugstores and drugstores/pharmacies. The measurements will be performed only at baseline (T0) before the intervention.
- Level of pharmacy staff satisfaction with the continuing education program. The measurements will be performed only on the intervention group pharmacy staff after completing the follow-up (T12).
- Level of satisfaction of patients or caregivers with ambulatory (outpatient) pharmacy services. The measurements will be performed at baseline (T0) before the intervention and 12 months (1 year) after completing the follow-up (T12)

### Data collection

Data will be collected between December 2018 and December 2019, conducted over 12 months of intervention delivery and follow-up. The data obtained in this study will be registered in an electronic database. The effectiveness of the continuing education program will be assessed before and after the intervention. The same questionnaires will be used in T0 and T12 for the pharmacy staff and the patients or caregivers. An additional measurement will be made for the intervention group 12 months after the end of the follow-up time in order to evaluate the effects of the educational intervention over time. All measuring instruments will be reviewed and adjusted by a group of 10 experts. Three different sessions will be carried out, in which the questionnaire will be presented, and the experts will be asked for their opinions regarding the relevance of the content of the items (content validity).

#### Community pharmacy staff competencies (knowledge, skills, and attitudes)

For the measurement of knowledge, a self-applied questionnaire of 50 multiple-choice questions (4 possible answers) with only one answer was constructed, and it will be categorized as correct or incorrect. The questionnaire will have the following topics: (1) pharmaceutical terminology and basic pharmacology, (2) pharmaceutical legislation, (3) technical and administrative management of the pharmacy, (4) communication and health education from the pharmacy, (5) good practices of dispensing and proper use of medicines, (6) pharmacovigilance from the pharmacy, and (7) pharmaceutical intervention on some common diseases in pharmacy (depression, anxiety disorder, sleep disorders, and pain management).

For the measurement of skills and attitudes, a self-applied questionnaire of 100 questions or performance criteria was constructed, which will be evaluated according to the frequency in which it will perform. For this purpose, a Likert scale will be established with four possible options: never (1), sometimes (2), usually (3), or always (4) that evaluate the following competencies—(a) provision of patient care: initial contact with the patient, medical prescription, medication needs, medication reconciliation, medication supply, use of guidelines and protocols, drug specifications, drug interactions, information about medications and patient education, additional activities, identification of drug-related problems, patient care, and evaluation of results; (b) personal: personal organization, practical communication skills, teamwork, and professionalism; (c) problem-solving: access related to information, knowledge, information analysis, and monitoring the patient; and (d) management and organization: clinical management and pharmacological safety, service provision, acquisition, and satisfaction of the services offered.

For the construction of the questionnaire, a search of reference documents, associations, and working groups in this area was carried out at an international level, focused on the guidelines and recommendations related to the proper functioning of the ambulatory pharmaceutical services and drugstores and drugstores/pharmacies. The international reference documents of the International Pharmaceutical Federation (FIP) and the World Health Organization [16, 17, 24] were used as sources of review, in which a minimum set of competencies was described within the different frameworks of professional performance. Additionally, different labor competency frameworks in different countries such as England, Ireland, and Australia [25–27] and the regulations in Colombia associated with the labor competencies of the pharmacy staff were reviewed. Additionally, a structured review was conducted on the measurement and evaluation of labor competencies, with the following MeSH terms: “Professional Competence” OR “Health Knowledge, Attitudes, Practice” AND “Pharmacists.” Finally, the results of the reviews were considered. The adjustment and revision of the instrument were carried out with a group of experts from the areas of healthcare pharmacy, research, and teaching. In total, 993 bibliographic references were identified with the search strategy, selecting nine articles that globally assessed competencies in 4 countries: Croatia, Australia, England, and Serbia [25–33].

#### Simulated patient technique

The simulated patient technique will be used to evaluate the skills and attitudes of the pharmacy staff in the dispensation and the information supply for the correct use of drugs with fiscalized substances. The simulated patients will be trained to represent and act coherently and accurately predetermined disease, specifically in pain management. Before carrying out the visits, the simulated patients who were previously trained would sign an informed consent in order to guarantee the confidentiality of the information collected. The pharmacy staff will not know at any time that the simulated patient is not real. A questionnaire will be constructed which will assess the following: (1) the drug dispensed, (2) the use of tools to provide information, and (3) the information provided on drug precautions and recommendations. The questionnaire will evaluate the minimum information that the pharmacy staff should provide to patients according to the indications, precautions, and recommendations for the use of the drug and the disease that will simulate (tramadol and lower back pain) [34, 35]. Both the information and the dispensing process must be carried out under the regulatory requirements.

#### Characteristics of the event and simulated patient

Patient with an average age of 20 years will go to the drugstores or drugstores/pharmacies with a medical formula of tramadol 100 mg/ml bottle 10 ml drops (10 drops/8 h), prescribed by a doctor, for a week-long back pain associated with physical exertion. There will be training performed for simulated patients. Additionally, a first dispensation will simulate (simulated patient will use tramadol for the first time). To guarantee the quality and validity of the data, one of the researchers will transport the simulated patients to all the visits and thus will validate the process and the storage of the data.

#### Characterization of the processes in the drugstores and drugstores/pharmacies

A self-designed questionnaire will be constructed from the requirements of Colombian pharmaceutical regulations and different bibliographical references [36, 37] to characterize the processes associated with the use the drugs with fiscalized substances, including compliance with the regulations, and to determine the level of risk perception with of use of these drugs in the pharmacy staff.

#### Implementation of the ambulatory (outpatient) pharmacy services

A questionnaire will be constructed from the requirements of Colombian pharmaceutical regulations and different bibliographical references [36, 37] for the measurement and degree of implementation of ambulatory (outpatient) pharmacy services.

#### Level of pharmacy staff satisfaction with the continuing education program

A questionnaire will be constructed from different bibliographical references [36, 38] to evaluate the satisfaction level of pharmacy staff about the continuing education program. A 5-point scale ranging from excellent, good, satisfactory, needs improvement, and unacceptable will be used in the questionnaire.

#### Level of satisfaction of patients or caregivers with the ambulatory (outpatient) pharmacy services

A questionnaire will be constructed from the requirements of Colombian pharmaceutical regulations and different bibliographical references [39, 40] to evaluate the information and education services on the appropriate use of drugs. The questionnaire will inquire about the following: (1) establishment information, (2) patient or caregiver information, (3) medication information, (4) assessment of the information and education service, and (5) qualification of the care received. The satisfaction of the care received in drugstores or drugstores/pharmacies will be evaluated with a 5-point scale ranging from excellent, very good, good, satisfactory, regular, and bad

To promote the retention of participants and complete follow-ups, the pharmacy staff will receive a physical certificate when he/she completes each module of the virtual course, which is part of the intervention (intervention group). Additionally, at the end of the follow-up time, all pharmacies of the intervention and control groups will receive a physical certificate that guarantees that said establishment participated in the study.

### Data management

Only one of the researchers involved in the trial will have access to the data entry. The data will be controlled by a second investigator to avoid errors in transcription. The data will be stored in a database. Those involved in this study will be the only ones with access to the information. We will back up the database every week in 2 different computers to protect the information. All the questionnaires will be carried out in physical format, except the questionnaire that evaluates the satisfaction of the continuing education program in the intervention group, which will be sent by means of an electronic survey, and the information will be stored online in Excel format.

### Statistical methods

Baseline and demographic characteristics will be analyzed descriptively. All primary and secondary outcomes will be compared in pre-post analyses and between-group comparisons. Comparisons for categorical variables will be conducted by using the chi-square test (or the Fisher exact test when appropriate). For continuous variables, we will use the independent sample *t* test (if the distribution is not normal, we will use the Mann-Whitney *U* test). Student’s *t* test will be used to compare means and odds ratios (ORs), and 95% confidence intervals (CIs) will be estimated as well. ORs will be used with the data reported from the knowledge questionnaire, to establish the association between receiving or not receiving the intervention and improving or not knowing. Spearman’s correlation will be used to identify the association for test and re-test reliability. Multivariable analyses will be performed to explain the association of multiple variables with the factors significantly related to the primary outcome (such as the job profile of the pharmacy staff, and others that will be considered as relevant variables). The McNemar tests will be used to analyze the difference in scores of the competency’s questionnaire of skills and attitudes and the scores of the knowledge questionnaire. All statistical tests will be computed using the Statistical Package for the Social Sciences (version 23; SPSS, Chicago, IL, USA) with significance defined as a *p* < 0.05.

### Data monitoring

The Trial Steering Committee (TSC) will be formed up of two researchers of trial, the general coordinator of the Coordinating Center of the Trial (CCT), and the administrative staff of the sponsor. The Pharmaceutical Promotion and Prevention research group will be the Coordinating Center of the Trial (CCT). This group is formed up of teachers, students, and graduates of pharmaceutical chemistry from the Faculty of Pharmaceutical and Food Sciences of the University of Antioquia. It will be in charge of the academic management and technical aspects of the study, also meeting spaces between researchers and sponsors. The person in charge of all aspects of the local organization, including the identification of possible recruits and consent, will be in charge of one of the members of the Pharmaceutical Promotion and Prevention research group, who will not be part of the study as a researcher.

The Data Monitoring Committee (DMC) will be the Technical Research Committee of the Research and Extension Center of the University of Antioquia, which is made up of teaching and administrative staff. The committee will perform the role of administrative management; the approval, control, and monitoring of financial resources; and the final compliance with the commitments of the trial. This committee will oversee the trial and request follow-up reports every 6 months, which will be done by two pharmacists of the Pharmaceutical Promotion and Prevention research group. A Stakeholder and Public Involvement Group (SPIG) will not be formed for this trial.

There are no planned interim analyses or “detention rules” specific to the study. Relevant agencies will be informed about the participation of pharmacy staff and pharmacies during their entire participation in the trial. We will adhere to international confidentiality and research practices and recommendations to monitor, audit, and administer the investigation. Due to the nature of the study, it is not necessary to make plans to collect, evaluate, report, and manage spontaneously requested and reported adverse events and other unwanted effects of trial interventions or behaviors. The periodic storage of the information in the database will show reports of the evaluation instruments, which will allow the monitoring of quality and data and generate reports according to the needs. The researchers involved in this study will be the only ones with access to the information. Every week, we will make a backup copy of the database on two different computers to protect the information.

## Limitations and potential bias

This study will use the simulated patient methodology as a tool to evaluate the skills and attitudes of customer service provided by pharmacy staff. This methodology has validity and reliability in pharmacy practice research [41]. To guarantee the quality of information, the simulated patients will receive training. Furthermore, a researcher will accompany the simulated patients on all visits to the drugstores and drugstores/pharmacies. The same researcher will make sure that the simulated patients complete the questionnaire immediately after each visit, thus controlling for common biases in this type of study. One possible limitation of our study is that the visits will not be recorded to minimize the possible biases associated with the collected information, as has been recommended by some systematic reviews [41].

## Discussion

We have planned this randomized trial to evaluate the effect of drugs with fiscalized substance (including controlled drugs) dispensation, health education, and pharmacovigilance continuing education programs in drugstores or drugstores/pharmacies. The intervention of this trial will be designed, developed, implemented, and evaluated, as a continuing education strategy to promote competencies in the proper utilization of drugs with fiscalized substances (including controlled drugs) in pharmacy staff, and therefore, it will contribute to reducing deficiencies in medical care. In Colombia, to our knowledge, this is the first controlled trial designed to assess pharmacy staff competencies.

Drugstores or drugstores/pharmacies play an essential role in the improvement of pharmacotherapy and patient outcomes, promotion of the rational use of drugs, and reduction of healthcare costs [42]. Besides, an employee eligible to work in an ambulatory (outpatient) pharmacy in Colombia is required to have one of the following educational levels (professional, technological, or technical). Besides, there are other empirical profiles called Drug Retailer (who does not have an educational level and is also called Drugstore Director). As a consequence, there is a large proportion of drugstores or drugstores/pharmacies that are managed by pharmacy staff who do not have the training and continuing education support [22, 43]. Recently, a cross-sectional, epidemiological study in Medellín (Colombia) drugstores or drugstores/pharmacies focused on the characterization of the use of drugs with fiscalized substances (including controlled drugs) and the pharmacy processes. Exactly 700 drugstores or drugstores/pharmacies were visited, and an equal pharmacy staff was interviewed. The most drugs with fiscalized substances dispensed without a valid medical prescription were amitriptyline 91.4% (640), tramadol 90% (630), and trazodone 60% (419). Around 60% of pharmacy staff do not have a technical or professional title, and their perception about the correct dispensing of drugs with fiscalized substances may be inadequate. A high percentage of the pharmacy staff perceives educational failures focused on the correct use of drugs [22].

Additionally, a systematic review describes different educational interventions with the support of ICTs and the effects on pharmaceutical knowledge and skills found. According to this review, they included 19 articles made mainly in developed countries. Most of the studies included in the review focused on evaluating the effect of continuing education programs on changing pharmacists’ knowledge and skills, and only one study evaluated the benefits of the program for patients or clients. The review authors conclude that there is insufficient evidence to support which methods from ICTs are the most effective and that it is necessary to intervene with robust, standardized, and validated methods [44].

A labor competence refers to a behavior that brings together different elements, performance criteria, and knowledge that determine the achievement, dominance, or fulfillment of the competence in a real work environment, which can be measured and evaluated, based on the combination of qualitative and quantitative research techniques, such as the observation and use of measuring instruments, respectively. Its measurement and evaluation should always be carried out jointly considering the combination of knowledge (what do you know?), skills (how do you do it?), and attitudes (how do you do it?). In this context, the pharmacy staff needs to improve their required competencies to contribute to the quality of drug dispensation, health education, and pharmacovigilance, in order to minimize medication errors and to help outpatients better manage their medicines. Continuing education, communication, and relationships with other health professionals are crucial to improving these competencies and, consequently, to achieve optimal patient medication management.

## Trial status

This study is currently in follow-up and intervention.

Protocol version: 0017102017MC. Registered on 28 November 2017.

Trial registration: NCT03388567.

Date of recruitment began on December 1, 2018.

Date of recruitment will be completed on December 31, 2019.

## Supplementary information

**Additional file 1.**

## Data Availability

The data sets generated and/or analyzed during the current study are not publicly available but are available from the corresponding author on reasonable request.

## Abbreviations

WHO: World Health Organization
FIP: Pharmaceutical International Federation
ICT: Information and Communication Technologies
CEP: Continuing education program
OR: Odds ratio
CIs: Confidence intervals
