# Supplementary material for: Evaluation of the effects of a drug with fiscalized substance dispensation, health education, and pharmacovigilance continuing education program in Colombia drugstores and drugstores/pharmacies: study protocol of a multicenter, cluster-randomized controlled trial
Source: Trials. 2020 Jun 19;21:545. doi: 10.1186/s13063-020-04481-1 (PMC7304186; doi:10.1186/s13063-020-04481-1)
Supplement: Supplementary file 1 — Additional file 1. [file 13063_2020_4481_MOESM1_ESM.docx]

**Annexed 1. Informed consent for patients**

**Introduction**: We are conducting a research study to assess the needs of patients and improve the proper use of drugs in the city of Medellín and the Metropolitan Area.

**Purpose**: We want to use this information to create strategies to improve the proper use of medications.

**Participation**: Next, I will read what the research consists of and ask if you want to participate voluntarily in the study. We are inviting you to participate in this study because we believe that you can provide us with useful information.

**Costs**: To participate in this study, you must NOT pay or assume any cost.

**Risks/Discomforts**: This is a without risk research. When you remember situations that make you feel bad, you have the right to end the interview or change your question. If you decide not to continue participating, this will not generate consequences.

**Rights**: Participation in the study is voluntary, it is your decision to talk to me. You can stop this interview at any time, and you can also refuse to answer any questions. This will not bring consequences or reprisals for you, or for your family, or for the staff pharmacy.

**Confidentiality**: The University of Antioquia and the study researchers guarantee that the information you provide us will be kept safe, and we will not use this information for purposes other than those required for the study. The individual information registered here will in no way be shared with any person or public or private entity. This way you can have all the freedom and confidence to answer the questions about the procedures, risks, benefits, and other matters related to the investigation, without fear of being identified.

**Signature patient or caregiver_______________________________________________**

**Name ___________________________________________________________________**

**Date ____________________________________________________________________**

**Name witness 1: _________________________________________________________**

**What is the relationship: ___________________________________________________**

**Name witness 2: __________________________________________________________**

**What is the relationship: ___________________________________________________**

**Annexed 2. Informed Consent for Pharmacy Staff**

**Introduction**: We are carrying out a research study to measure the effect of a training education program on dispensing, health education, and pharmacovigilance services of the drugs with substances fiscalized in drugstores of Medellin and the Metropolitan Area.

**Purpose**: We want to use this information to create strategies that allow improving activities and services of dispensing, health education, and pharmacovigilance.

**Costs**: To participate in this study and answer the questionnaires, you must NOT pay or assume any cost.

**Risks/Discomforts**: This is a without risk research. If you decide not to continue participating, this will not generate consequences.

**Rights**: Participation in the study is voluntary; it is your decision to speak with the researchers. You have the right to withdraw from the study at any time.

**Confidentiality**: The University of Antioquia and the study researchers guarantee that the information you provide us will be kept safe, and we will not use this information for purposes other than those required for the study. The individual information registered here will in no way be shared with any person or public or private entity. This way you can have all the freedom and confidence to answer the questions about the procedures, risks, benefits, and other matters related to the investigation, without fear of being identified

**Compensation**: By participating in the study, you will not receive any kind of financial compensation. However, you and your drugstore will receive physical certificates from the University of Antioquia in which your participation in this study will be highlighted.

**Consent to Participate**: I have read this form, and I understand it completely. All my questions about this form or the study were answered satisfactorily.

**Signature staff pharmacy_______________________________________________**

**Name ___________________________________________________________________**

**Date ____________________________________________________________________**

**Name witness 1: __________________________________________________________**

**What is the relationship: ___________________________________________________**

**Name witness 2: __________________________________________________________**

**What is the relationship: ___________________________________________________**

**Annexed 3. Confidentiality Agreement and Informed Consent for Simulated Patients**

**Introduction**: We are carrying out a research study to evaluate the skills and attitudes of staff pharmacists of drugstores and drugstores/pharmacies in the drug with substances fiscalized dispensing in Medellin and the Metropolitan Area.

**Purpose**: We want to use this information to create strategies that allow improving activities and services of dispensing, health education, and pharmacovigilance.

**Costs**: To participate in this study and answer the questionnaires, you must NOT pay or assume any cost.

**Risks/Discomforts**: This is a without risk research. If you decide not to continue participating, this will not generate consequences.

**Rights**: Participation in the study is voluntary; it is your decision to speak with the researchers. You have the right to withdraw from the study at any time.

**Compensation**: By participating in the study, you will not receive any kind of financial compensation.

**Consent to Participate**: I have read this form, and I understand it completely. All my questions about this form or the study were answered satisfactorily.

**Confidentiality Agreement:** It is relevant and necessary that you guarantee that the information that you help us collect about the pharmacy staff and drugstores that you will visit will in no way be shared with any person or public or private entity, in order to guarantee confidentiality and information.

**Signature staff pharmacy_______________________________________________**

**Name ___________________________________________________________________**

**Date ____________________________________________________________________**

**Name and signature witness 1: ______________________________________________**

**What is the relationship: ___________________________________________________**

**Name and signature witness 2: ______________________________________________**

**What is the relationship: ___________________________________________________**

**Annexed 4. Ambulatory drugs with fiscalized substances in Colombia**

| Alprazolam |
| --- |
| Amisulpride |
| Amitriptyline |
| Bromazepam |
| Brotizolam |
| Buprenorphine |
| Cinnarizine |
| Clobazam |
| Clomipramine |
| Clonazepam |
| Clozapine |
| Codeine |
| Diazepam |
| Diphenoxylate |
| Dihydrocodeine |
| Dinoprostone - prostaglandin E2 |
| Dronabinol |
| Ephedrine |
| Ergotamine |
| Phenobarbital |
| Haloperidol |
| Hydrocodone |
| Hidromorfona |
| Imipramine |
| Ketotifen |
| Levomepromazine |
| Lorazepam |
| Maprotiline |
| Meperidine |
| Metadona |
| Methylphenidate |
| Midazolam |
| Morphine |
| Nalbuphine |
| Oxycodone |
| Oxytocin |
| Pipothiazine |
| Pseudoephedrine |
| Sulpiride |
| Tapentadol |
| Thioridazine |
| Tramadol |
| Trazodone |
| Triazolam |
| Trifluoperazine |
| Zolpidem |
